# Two new sympatric species of *Phrynopus* (Anura: Strabomantidae) from the Elfin Forests of Cordillera de Yanachaga in central Peru

**DOI:** 10.7717/peerj.20250

**Published:** 2025-10-30

**Authors:** Pablo Venegas, Luis Alberto García Ayachi, Lesly Lujan, Vilma Duran, Ana Motta

**Affiliations:** 1 Rainforest Partnership, Austin, TX, United States of America; 2 Instituto Peruano de Herpetología (IPH), Lima, Peru; 3 Biodiversity Institute and Natural History Museum, University of Kansas, Lawrence, KS, United States of America

**Keywords:** Andes, Anura, Amphibia, Terrarana, Phylogeny, Singleton species

## Abstract

We describe two new sympatric species of the terrestrial-breeding genus *Phrynopus* (Anura: Strabomantidae) from the elfin forest at 3,280 m a.s.l. in the Cordillera de Yanachaga, Yanachaga-Chemillén National Park, central Peru. Integrating molecular and morphological evidence, we aim to confirm their recognition as new species and assess their generic placement and relationships within *Phrynopus*. We infer a Maximum-Likelihood phylogeny from five loci (12S, 16S, COI, RAG1, TYR; 4,271 bp of concatenated mtDNA and nuDNA fragments) for 97 terminals, including three representing the new taxa. *Phrynopus* was recovered as monophyletic, and both new species were placed within a strongly supported subclade that includes *P. apumantarum*, *P. badius*, *P. barthlenae*, *P. bracki, P. bufoides*, *P. horstpauli*, *P. inti*, *P. kauneorum*, *P. miroslawae*, *P. pesantesi*, *P. sancristobali*, *P. tautzorum,* and *Phrynopus* sp. The two new species are not recovered as close sisters but as distinct lineages within this subclade. One of the new species is medium-sized, distinguished by small tubercles on the upper eyelids, tubercles on the heel, a row of tubercles along the outer edge of the tarsus, and red coloration on the groin, thighs, and concealed surfaces of the shanks. The other new species lacks heel and tarsal tubercles and is characterized by its striking black coloration on the groin and hidden surfaces of the hind limbs. Both new species are currently known only from the type locality, where they occur in sympatry with *P. miroslawae* and *P. tribulosus*. The discovery of these narrowly distributed species in the Yanachaga-Chemillén National Park, coupled with habitat alteration near the boundaries of the park, highlights the urgent need for effective protection of elfin-forest habitats in the Cordillera de Yanachaga.

## Introduction

Terrestrial breeding frogs of the family Strabomantidae are highly diverse, with more than 800 species distributed in tropical and subtropical South America and lower Central America (Frost, 2025). Species of this group deposit their eggs in terrestrial sites where they undergo direct development, lacking the aquatic tadpole stage. This mode of life history, not associated with aquatic environments, is responsible for their success in inhabiting a variety of environments, including cloud forests, lowland forests, dry forest and humid grasslands (Duellman & Lehr, 2009; Frost, 2025; Hedges, Duellman & Heinicke, 2008). Strabomantid species comprise about half of all species of frogs known to inhabit Peru, where they are distributed in 12 genera (*Bryophryne*, *Lynchius*, *Microkayla*, *Niceforonia*, *Noblella, Oreobates, Phrynopus*, *Phyllonastes, Pristimantis*, *Qosqophryne*, *Strabomantis* and *Yunganastes*), occupying a variety of habitats such as Pacific dry forest, humid lowland tropical forests, montane forests, puna and paramo (Duellman & Lehr, 2009; Frost, 2025; Von May et al., 2024).

The high Andean strabomantid frogs share a similar external morphology (the “phrynopoid” morphology) that led them to be historically considered part of a single natural group, the genus *Phrynopus* (De la Riva, 2020). *Phrynopus* was re-erected to include 14 species of small frogs, with short limbs and digits without discs, broadly distributed in the high Andes from Colombia to Bolivia (Lynch, 1975). Subsequently, the genus experienced a rapid increase in species described and drastic changes in its composition (De la Riva, 2007; De la Riva, Chaparro & Padial, 2008; Lehr, 2006).

The monophyly of *Phrynopus* sensu Lynch (1975) was rejected by molecular phylogenetic analyses, revealing a scenario where high-elevation lineages have independent origins (Hedges, Duellman & Heinicke, 2008). The non-monophyletic *Phrynopus* was split into a number of genera, and the genus *Phrynopus* was restricted to a clade of 21 species that occur in upper humid forests and grasslands of the Cordillera Oriental in Peru (Hedges, Duellman & Heinicke, 2008). In the following decade, 16 new species of *Phrynopus* were described and the redefined genus faced another increase in its number of species (Chaparro, Padial & De la Riva, 2008; Chávez et al., 2015; Lehr, Moravec & Cusi, 2012; Lehr & Rodríguez, 2017; Lehr et al., 2017; Mamani & Malqui, 2014; Rodríguez & Catenazzi, 2017; Trueb & Lehr, 2008; Venegas et al., 2018; Von May, Lehr & Rabosky, 2018). With *P. curator* and *P. nicoleae* being considered synonyms of *P. tribulosus* (Von May, Lehr & Rabosky, 2018), *P. ayacucho* (Chaparro et al., 2007) being transferred to the genus *Oreobates* (Padial et al., 2012), and the recent description of *P. apumantarum*, *P. remotum*, and *P. sancristobali*, the genus now comprises 37 species, distributed in the Cordillera Oriental and Central of Peru, restricted to a region between 6° and 13° of latitude (Chávez, García-Ayachi & Catenazzi, 2020; Chávez, García-Ayachi & Catenazzi, 2023; Díaz, Mamani & Catenazzi, 2023; Venegas et al., 2018). Species have been recorded at elevations between 2,600–4,490 m, and most of them show very restricted ranges, both horizontally and vertically (Chávez, García-Ayachi & Catenazzi, 2023; Rodríguez & Catenazzi, 2017).

Even with the discovery of numerous new species resulting from recent fieldwork, the diversity of species of *Phrynopus* is still considered underestimated, as many remote regions of the slopes of the Andes in Peru have not yet been explored and probably are inhabited by additional microendemic taxa (De la Riva et al., 2018). The Yanachaga-Chemillén National Park in the Departamento de Pasco of Peru has a remarkable diversity of amphibians (41 species have been recorded for the area, including undescribed species; Angulo et al., 2016), and fieldwork in the area has led to the discovery of new species of strabomantid frogs, including new species of *Phrynopus* (Duellman & Hedges, 2005; Duellman & Hedges, 2008; Hedges, 1990; Lehr, Moravec & Cusi, 2012; Lehr et al., 2017). Fieldwork in this region conducted by some of the authors of this study revealed the existence of two unnamed species of *Phrynopus*. We use phylogenetic analyses of nuclear and mitochondrial genes to assess their phylogenetic relationships and combine morphological and molecular data to support the recognition of the two species described in this study.

## Materials & Methods

### Morphology, voucher specimens, and permits

Character definition and terminology, and the descriptive scheme of the diagnosis follow that of Duellman & Lehr (2009). Specimens were measured with digital calipers to the nearest 0.1 mm. The following measurements were recorded: snout–vent length (**SVL**); tibia length (**TL**); foot length (**FL**, measured from the proximal edge of the inner metatarsal tubercle to the tip of Toe IV); head length (**HL**, taken obliquely from the jaw angle to the snout tip); head width (**HW**, at the jaw angle); eye diameter (**ED**); interorbital distance (**IOD**); upper eyelid width (**EW**); internarial distance (**IND**); and eye–nostril distance (**E–N**, the straight-line distance between the anterior corner of the orbit and the posterior margin of the external naris). Fingers are numbered from I–IV, starting preaxially. To compare relative lengths, Fingers I and II were pressed together, while Toes III and V were each placed against Toe IV. Specimens were preserved in 10% formalin and stored in 70% ethanol. Tissue samples were collected before fixing specimens with formalin and preserved in 95% ethanol. Specimens were sexed externally by the presence or absence of vocal slits and internally by the condition of the gonads. All specimens were deposited in the herpetological collection of the Centro de Ornitología y Biodiversidad (CORBIDI), Lima, Peru. Institutional abbreviations follow Sabaj (2020). We obtained our research permit through the Dirección General Forestal y de Fauna Silvestre, Ministerio de Agricultura y Riego, Peru, which issued the Contrato de Acceso Marco a Recursos Genéticos, numbered 359-2013-MINAGRI-DGFFS-DGEFFS. Our research was approved by the Institutional Animal Care and Use Committee of University of Kansas (AUS 279-01). Specimens examined are listed in Appendix S1.

### DNA extraction and sequencing

We extracted total DNA from ethanol preserved tissues, following standard high-salt protocol adapted for microcentrifuge tubes (Lyra, Haddad & De Azeredo Espin, 2017; Maniatis, Fritsch & Sambrook, 1982). We amplified two mitochondrially encoded gene fragments: one including the partial sequences of 12S rRNA, tRNA-val and 16S rRNA genes (H1 fragment) and a fragment of the cytochrome c oxidase I (COI); and two nuclear genes: partial sequences of tyrosinase (TYR), and partial sequences of recombination activating 1 (RAG1). Amplifications were carried out in a 22 µl reaction using Ampliqon Taq DNA Polymerase Master Mix (Ampliqon A/S, Odense M, Denmark), with primers listed in Table S1. For the mitochondrial genes we followed polymerase chain reactions (PCR) conditions described in Lyra, Haddad & De Azeredo Espin (2017) (set-up reaction (UP reaction) protocol). For nuclear genes we used the following PCR cycling protocol: 3 min of denaturation at 95 °C, followed by 45 cycles of 20 s of denaturation at 95 °C plus 20s of annealing at 56 °C plus 1 min of extension at 68 °C, followed by 3 min of final extension at 68 °C, and stored at 12 °C. PCR products were purified using an enzymatic reaction containing 1 unit of Exonuclease I and 0.5 unit of Alcaline phosphatase (Thermo Fisher Scientific Inc.) and were sent to Macrogen, Inc., Seoul, Repuiblic of Korea, for sequencing. Sequence files were checked for quality and contigs were assembled using Geneious R11 (Biomatters).

### Phylogenetic analysis

We used phylogenetic trees to assess generic assignment and investigate the relationship of the focal samples. We supplemented our sequences with sequences of the mitochondrial 12S rRNA and partial sequence of 16S rRNA genes, and the protein-coding gene cytochrome c oxidase subunit I (COI) as well as nuclear genes recombination-activating gene 1 (RAG1) and tyrosinase precursor (TYR) available on GenBank belonging to species of *Phrynopus*. One new species is represented by the terminal CORBIDI 7379, and the other one by the terminals CORBIDI 7382 and CORBIDI 7385. Our ingroup sample includes 63 terminals of *Phrynopus* representing 24 nominal species, two unnamed species (*Phrynopus* spI and *Phrynopus* sp. of Von May, Lehr & Rabosky, 2018), and the two species we name herein. As outgroups we included one terminal per species of the related genera *Lynchius* (*n* = 8) and *Oreobates* (*n* = 25) and rooted all our analyses with the distantly related species *Haddadus binotatus* (Padial, Grant & Frost, 2014). Specimen voucher numbers for newly produced sequences and GenBank accession numbers for all sequences used in this study are listed in Appendix S2.

We performed multiple sequence alignments in MAFFT online v7 using the global instreaming iterative (G-INS-i) strategy, which is considered appropriate for alignments that consist of large numbers of sequences (Katoh & Standley, 2013).

We generated Maximum Likelihood (ML) phylograms using IQ-TREE v1.6 (Nguyen et al., 2015) from the concatenated sequence of the five gene fragments included in our dataset. We determined the best-fit substitution model for each gene *via* ModelFinder, implemented within IQ-TREE (Kalyaanamoorthy et al., 2017) and performed a partitioned analysis according to codon position within the protein-coding genes (COI, RAG1, and TYR). We calculated branch support with 10,000 bootstrap replicates using the Ultrafast Bootstrapping algorithm (Hoang et al., 2018). Alignments, script, and output files, including partitions and trees, are available as supplementary files.

### Nomenclatural act

The electronic version of this article in Portable Document Format (PDF) will represent a published work according to the International Commission on Zoological Nomenclature (ICZN), and hence the new names contained in the electronic version are effectively published under that Code from the electronic edition alone. This published work and the nomenclatural acts it contains have been registered in ZooBank, the online registration system for the ICZN. The ZooBank LSIDs (Life Science Identifiers) can be resolved and the associated information viewed through any standard web browser by appending the LSID to the prefix http://zoobank.org/. The LSID for this publication is: urn:lsid:zoobank.org:pub:513EDEF4-3F07-4097-8902-8DFED468E4C3. The online version of this work is archived and available from the following digital repositories: PeerJ, PubMed Central SCIE and CLOCKSS.

## Results

### Phylogenetic relationships

**Figure 1:**
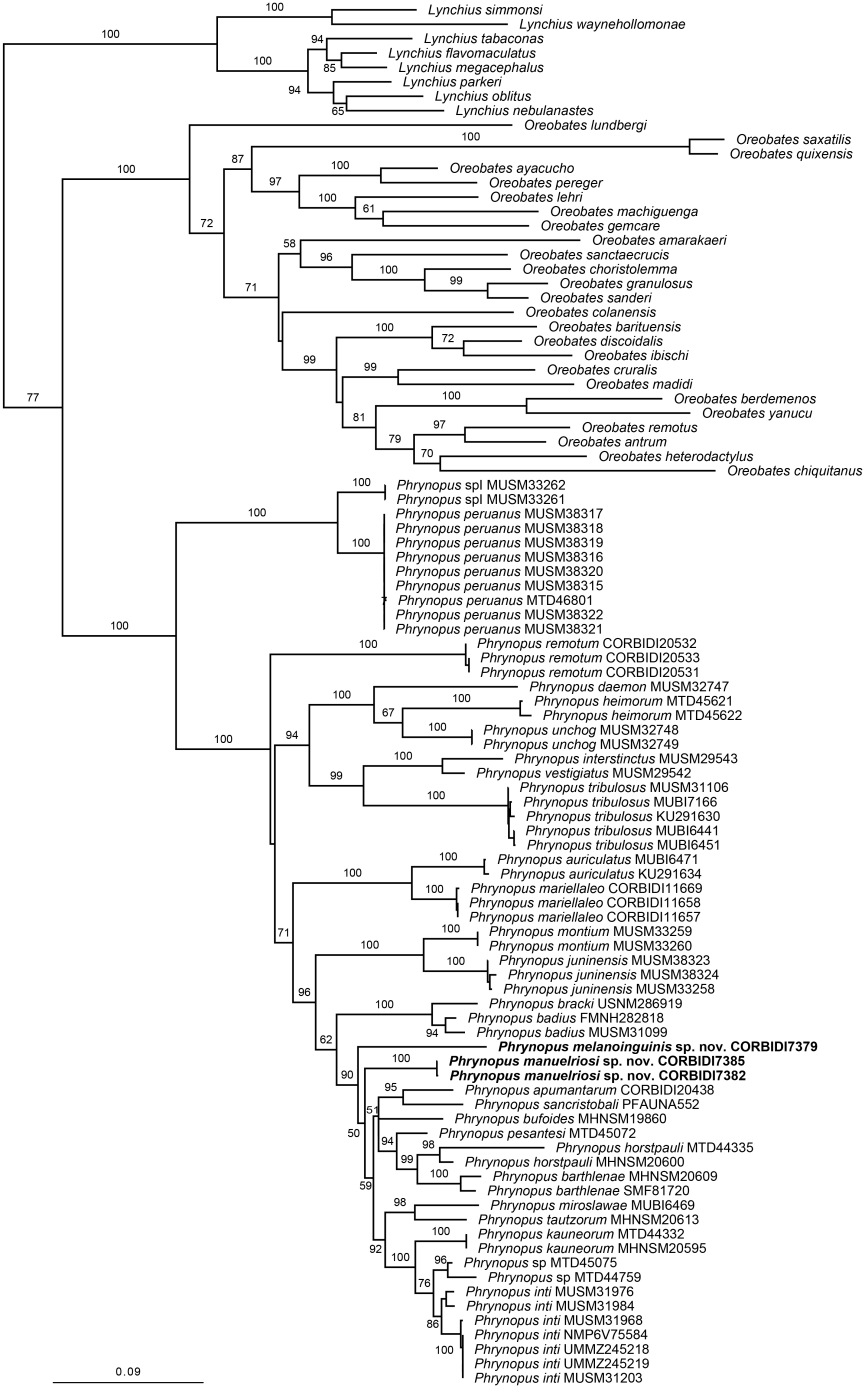
Phylogenetic tree resulting from the analysis of data sets of 4,271 aligned bp and composed of the mitochondrial genes 12S, 16S, and COI, and fragments of the nuclear protein-coding genes RAG1 and TYR. Maximum likelihood optimal tree (log likelihood −35,013.622762) and bootstrap node values. Asterisks represent values of 100.

The optimal similarity-alignment of our concatenated dataset comprises 4,271 character columns for 97 terminals. Our analysis recovered the genera *Phrynopus* as monophyletic, with 100% bootstrap support and supported the placement of the two new species in the genus *Phrynopus* (Fig. 1). The two new species are recovered as part of a highly supported clade (90%) that includes *P. apumantarum*, *P. badius*, *P. barthlenae*, *P. bracki, P. bufoides*, *P. horstpauli*, *P. inti*, *P. kauneorum*, *P. miroslawae*, *P. pesantesi*, *P. sancristobali*, *P. tautzorum* and *Phrynopus* sp. The phylogenetic position and morphological distinctiveness of the newly collected specimens support the description of the two new species, which we name and diagnose below.

**Table:** 

**Figure 2:**
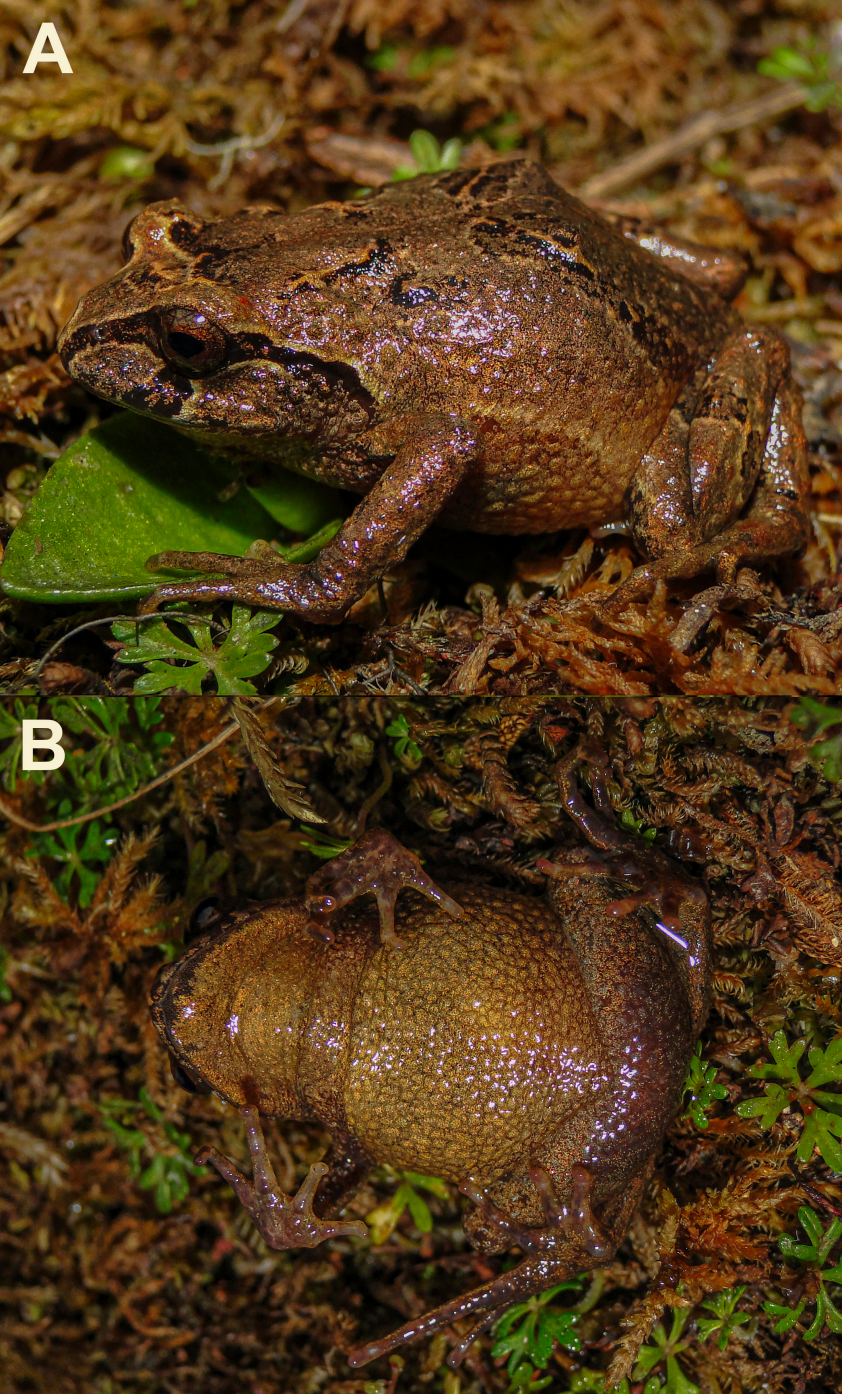
Holotype of *Phrynopus manuelriosi* sp. nov. (CORBIDI 7385; SVL 26.1 mm) in life. (A) Dorsolateral, and (B) ventral views.

**Figure 3:**
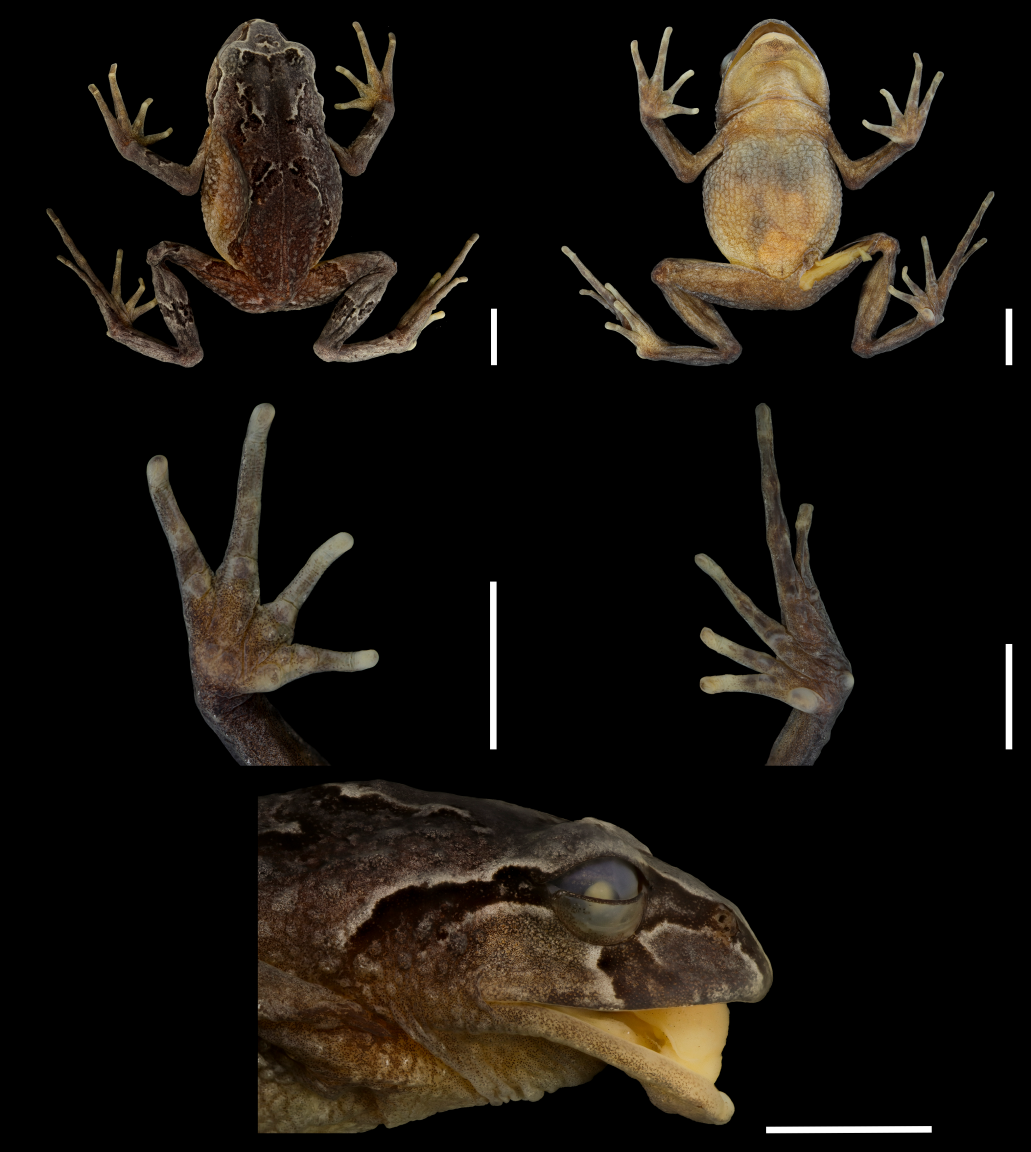
Preserved holotype of *Phrynopus manuelriosi* sp. nov. (A) Dorsal view, (B) ventral view, (C) palm, (D) sole, and (E) head in lateral view. Scale five mm. Photographs by LAGA.

**Figure 4:**
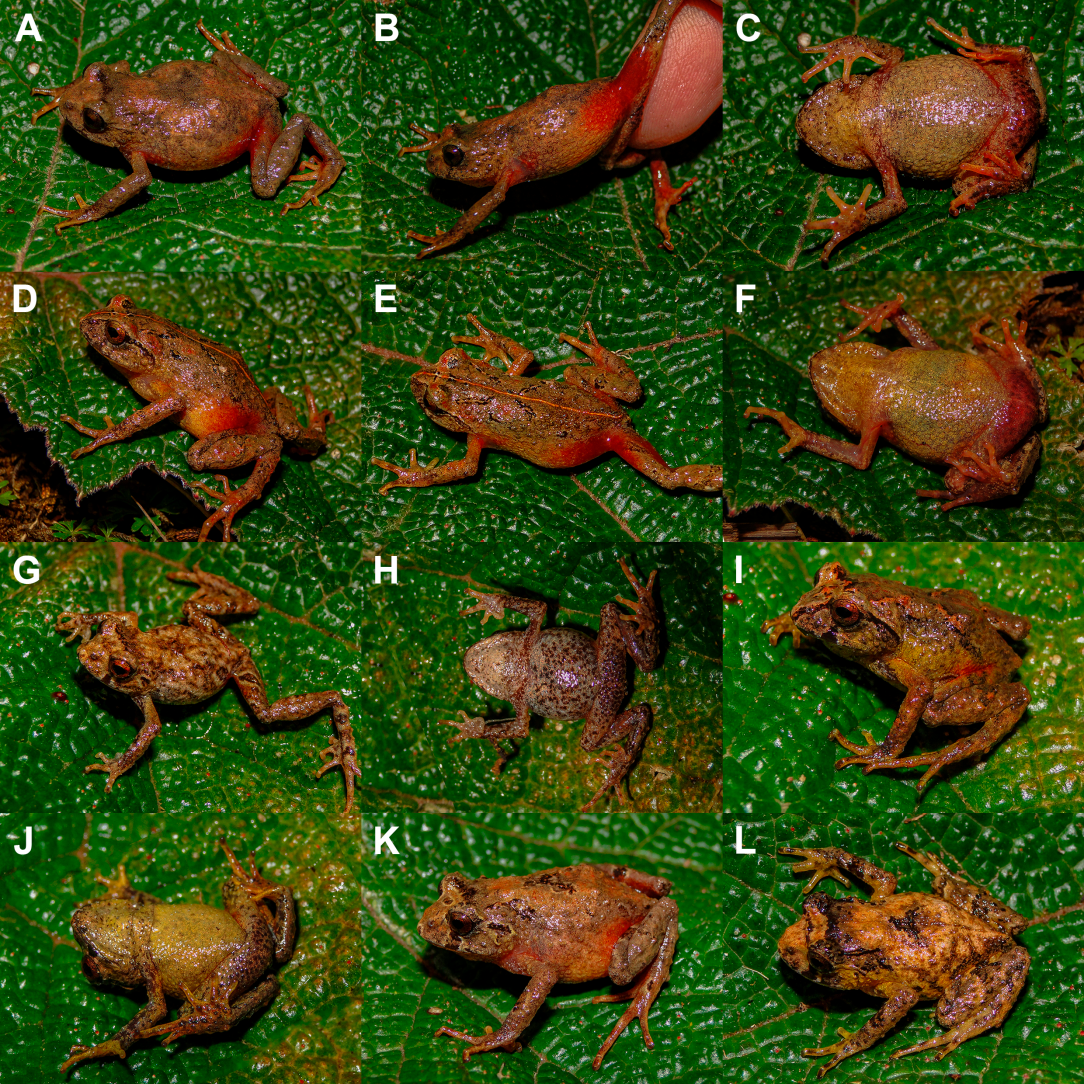
Paratypes of *Phrynopus manuelriosi* sp. nov. in life. (A) Dorsolateral (B) lateral (showing the groin and anterior surface of thigh), and (C) ventral views of CORBIDI 7380 (SVL 25.6 mm); (D) dorsolateral, (E) dorsal (showing a fine pale middorsal band, red groin, and the red anterior and posterior surface of thigh), and (F) ventral views of CORBIDI 7383 (SVL 27.1 mm); (G) dorsolateral and (H) ventral views of male paratype CORBIDI 7390 (SVL 13.2 mm); (I) dorsolateral and (J) ventral views of male paratype CORBIDI 7386 (SVL 17.4 mm); (K) dorsolateral view of female CORBIDI 7387 (SVL 19.4 mm); (L) dorsolateral view of male CORBIDI 7381 (SVL 16.1 mm).

***Phrynopus manuelriosi* ** sp. nov.****
urn:lsid:zoobank.org:act:86453AE1-9A6A-4ABD-9681-CF9E372B877D
Figs. 2–4; Table 1

### Holotype

CORBIDI 7385 (Fig. 2), adult female, Santa Bárbara, Distrito de Huancabamba, Provincia de Oxapampa, Departamento de Pasco, Peru, (10°20′29.1″S, 75°38′27.1″W, 3,280 m a.s.l.), collected by Pablo J. Venegas, Vilma Duran, Caroll Z. Landauro, and Lesly Lujan, on 25 August 2010.

### Paratypes (9)

Nine specimens in total, four adult females (CORBIDI 7380, 7382–83, 7387) and five adult males (CORBIDI 7381, 7386, 7388–90), same data as the holotype.

### Diagnosis

A species in the genus *Phrynopus* characterized by (1) skin on dorsum shagreen with scattered low tubercles, more abundant and prominent on flanks and hind limbs; usually bearing interorbital fold, \/-shaped fold on scapular region, and /\-shaped fold on the middle of dorsum; skin on venter areolate; groins smooth or weakly areolate; dorsolateral folds absent; supratympanic fold conspicuous and long, slightly curved above the tympanic region; discoidal fold present only as thoracic fold or completely absent; (2) tympanic membrane and annulus absent; (3) snout moderately short, bluntly rounded in dorsal view and in profile; (4) upper eyelid bearing small tubercles, narrower than IOD; cranial crests absent; (5) vomerine teeth absent; (6) vocal slits present and nuptial pads absent; vocal sac absent; (7) Finger I shorter than Finger II; tips of fingers rounded and narrow; (8) fingers lacking lateral fringes; subarticular tubercles small and rounded in dorsal view, and flat on lateral view; (9) ulnar tubercle present, more evident in males; (10) heel bearing one or two subconical tubercles and outer edge of tarsus bearing a row of broad conical tubercles; inner tarsal fold absent; (11) inner metatarsal tubercle ovoid, about equal in size to rounded outer metatarsal tubercle; subarticular tubercles small and rounded in dorsal view, flat on lateral view; supernumerary plantar tubercles present; (12) toes lacking lateral fringes; webbing absent; Toe V longer than Toe III; tips of toes rounded; (13) in life, dorsum of head, body, and limbs pale brown, yellowish-brown or grayish-brown with dark brown markings; groin, anterior and posterior surface of thighs, and concealed surface of shanks red; ventral surface yellowish-brown, grayish-brown or yellow with or without dark brown flecks on the throat and belly; iris red with black reticulations; (14) SVL in five males 11.3–17.4 mm, in five females 19.4–27.1 mm.

### Comparisons

Among the 37 described species of *Phrynopus*, only *P. badius, P. bracki*, *P. daemon*, *P. heimorum*, *P. inti*, *P. paucari*, *P. peruanus, P. unchog* and *P. vestigiatus* show reddish coloration in the groins (Duellman & Lehr, 2009; Lehr, 2001; Lehr, Moravec & Cusi, 2012; Lehr & Oroz, 2012; Lehr & Rodríguez, 2017). *Phrynopus manuelriosi* sp. nov. differs from *P. badius, P. bracki*, *P. inti*, *P. paucari* and *P. vestigiatus* by having uniformly red groin (groin dark brown with bright orange flecks in *P. badius*; brown with red spots in *P. bracki*; pale grayish with salmon-colored flecks in *P. inti*; greenish yellow with diffuse salmon blotches in *P. paucari*; and dark brown with red well-defined blotches in *P. vestigiatus*). Of the species sharing a uniformly reddish coloration in the groin, *P. manuelriosi* sp. nov. can be distinguished by the presence of heel tubercle (absent in *P. daemon*, *P. heimorum*, and *P. peruanus*), tarsal tubercles (absent in *P. heimorum* and *P. peruanus*), and eyelid tubercles (absent in *P. daemon*, *P. heimorum*, *P. peruanus*, and *P. unchog*). Moreover, *P. manuelriosi* sp. nov. lacks tympanic membrane and annulus, which are present in *P. peruanus*.

The presence of tubercles on the heel and outer edge of tarsus is uncommon in the genus *Phrynopus*. Only six species (*P. bracki, P. dagmarae, P. kotosh, P. oblivious, P. tribulosus* and *P. vestigiatus*) share the presence of tubercles on the heel and outer edge of tarsus. *Phrynopus manuelriosi* sp. nov. differs from *P. bracki* and *P. dagmarae* by having tubercles on the upper eyelids (absent in *P. bracki* and *P dagmarae*), and *P. dagmarae* has the Toe V shorter than Toe III, while the Toe V is larger than Toe III in the new species. *Phrynopus dagmarae, P. kotosh* and *P. oblivius* also differ by lacking \/-shaped fold in the scapular region and a /\-shaped fold on the middle of the dorsum. Moreover, *P. manuelriosi* sp. nov. lacks dorsolateral folds, whereas dorsolateral folds are present in *P. dagmarae* (continuous), *P. kotosh* (discontinuous), and *P. vestigiatus* (prominent and undulated). *Phrynopus tribulosus* has Toe V equal or slightly shorter than Toe III (Von May, Lehr & Rabosky, 2018), while in *P. manuelriosi* sp. nov. the Toe V is longer than Toe III. In addition, *P. barthlenae* and *P. miroslawae* have tubercles on the heel but lack tubercles on the outer edge of tarsus (present in *P. manuelriosi* sp. nov.) and Toe V is shorter than Toe III in *P. barthlenae* and equal in length in *P. miroslawae*, respectively, while *P. manuelriosi* sp. nov. has Toe V longer than Toe III.

*Phrynopus horstpauli* has a habitus similar to *P. manuelriosi* sp. nov., also being found in leaf and branches of the understory (Duellman & Lehr, 2009; Lehr, Köhler & Ponce, 2000) and sharing the slender limbs and relative long narrow fingers and toes. *Phrynopus manuelriosi* sp. nov. can be easily distinguished from *P. horstpauli* by having one or two subconical tubercle on the heel and a row of conical tubercles on the outer edge of the tarsus (absent in *P. horstpauli*), and a smaller size with a SVL of 11.3 to 17.4 mm in males and 19.4 to 27.1 mm in females (17.7 to 25.6 mm in males and 30.8 to 39.7 mm in females of *P. horstpauli*).

*Phrynopus melanoinguinis* sp. nov., described bellow, occurs in sympatry with *P. manuelriosi* sp. nov. and it can be easily distinguished by lacking heel and tarsal tubercles (present in *P. manuelriosi* sp. nov.), and by having dorsolateral fold (absent in *P. manuelriosi* sp. nov.).

Due to the variable coloration of *P. manuelriosi* sp. nov., we consider it possible to confuse it with *Noblella duellmani*, a geographically close species. *Noblella duellmani* occurs in Departamento de Pasco, at elevations between 2,900 and 3,500 m, the same elevational range of *P. manuelriosi* sp. nov. *Noblella duellmani* can be easily distinguished from *P. manuelriosi* sp. nov. by having Toe V shorter than Toe III (Toe V longer than Toe III in *P. manuelriosi* sp. nov.), tips of digit slightly expanded and those of Toes III–V slightly acuminate (tips of toes narrow and rounded in *P. manuelriosi* sp. nov.), and skin of belly smooth (areolate in *P. manuelriosi* sp. nov.).

**Table 1:** Variation of measurements (in mm) and proportions of the type series of *Phrynopus manuelriosi***sp. nov**. Abbreviations are as follow: SVL, snout–vent length; TL, tibia length; FL, foot length; HL, head length; HW, head width; ED, eye diameter; IOD, interorbital distance; EW, upper eyelid width; IND, internarial distance, and E-N, eye–nostril distance.

**Measurements and proportions**	**Females** ***N = 5***	**Males** ***N = 5***
**SVL**	19.4–27.1 (24.8 ± 3.1)	11.3–17.4 (14.3 ± 2.4)
**TL**	9.7–11.9 (11.2 ± 0.8)	6–8.7 (7.2 ± 1.1)
**FL**	9.9–14.1 (12.2 ± 1.5)	5.9–9.5 (7.4 ± 1.5)
**HL**	7.8–10.2 (9.2 ± 0.9)	4.4–7.2 (5.7 ± 1.2)
**HW**	7.6–9.3 (8.7 ± 0.6)	3.9–6.6 (5.3 ± 1.7)
**ED**	2.7–3.3 (2.9 ± 0.3)	1.7–2.3 (2.2 ± 0.2)
**IOD**	2.7–3.1 (2.9 ± 0.1)	1.8–2.5 (2.6 ± 0.2)
**EW**	1.7–2.6 (2.1 ± 0.3)	1.4–1.9 (1.6 ± 0.1)
**IND**	1.5–1.9 (1.7 ± 0.1)	1.0–1.7 (1.2 ± 0.2)
**E–N**	1.6–1.9 (1.7 ± 0.1)	1.0–1.3 (1.1 ± 0.1)
**TL/SVL**	0.4–0.5 (0.4 ± 0.3)	0.4–0.5 (0.5 ± 0.2)
**FL/SVL**	0.4–0.5 (0.4 ± 0.4)	0.4–0.5 (0.5 ± 0.4)
**HL/SVL**	0.3–0.4 (0.3 ± 0.2)	0.3–0.4 (0.6 ± 0.2)
**HW/SVL**	0.3–0.3 (0.3 ± 0.3)	0.3–0.3 (0.8 ± 0.1)
**HW/HL**	0.8–1.4 (0.9 ± 0.7)	0.8–1.2 (0.8 ± 0.5)
**E–N/ED**	0.5–0.7 (0.5 ± 0.7)	0.4–0.7 (0.4 ± 0.1)
**EW/IOD**	0.6–0.8 (0.7 ± 0.9)	0.6–0.9 (0.8 ± 0.1)

### Description of holotype

Adult female (Figs. 2 and 3); body moderately robust; head about as wide as body, nearly as long as wide; snout bluntly rounded in dorsal view and in profile; canthus rostralis slightly curved in dorsal view, rounded in profile; loreal region nearly flat; lips rounded; nostrils barely protuberant, directed laterally; internarial region barely depressed; top of head flat; width of upper eyelid narrower than interorbital distance (EW/IOD 0.84); eye large, its diameter much greater than its distance from nostril (E-N/ED 0.70); tympanic membrane and annulus absent; supratympanic fold distinct, angling posteroventrally from point behind the tympanic region close to the arm insertion; postrictal tubercles present, rounded. Tongue longer than broad, notched posteriorly, posterior half free; choanae small, round, not concealed by palatal shelf of maxillary; dentigerous processes of vomers absent.

Forelimb slender; ulnar tubercles low, diffuse; palmar tubercle low, round, about same size as thenar tubercle; subarticular tubercles distinct, small and rounded in dorsal view, and flat in lateral view; supernumerary tubercles present, weakly defined; fingers slender and long, lacking lateral fringes; relative lengths of fingers I < II < IV < III; tips of fingers narrow, rounded, lacking circumferential grooves. Hind limb long and slender; heel bearing two small subconical tubercles and tarsus bearing three low conical tubercles, broad at their base; inner tarsal fold present on distal half of tarsus; inner metatarsal tubercle elevated, round, about twice the size of subconical outer metatarsal tubercle; subarticular tubercles distinct only at the base of toes, small and rounded in dorsal view, flat in lateral view; supernumerary tubercles present, distinct; toes slender, lacking lateral fringes; relative lengths of toes I < II < III < V < IV; tips of toes narrow, rounded, lacking circumferential grooves. Skin on dorsum shagreen with scattered low tubercles posteriorly, bearing an interorbital fold, a V-shaped fold on scapular region, and a / \-shaped fold on the sacrum; flanks and hind limbs tuberculate; upper eyelids bearing low rounded tubercles; skin on venter areolate; thoracic fold present; skin ventral and ventrolateral to cloaca granular.

Measurements (in mm) and proportions of holotype: SVL 26.1; TL 11.5; HW 9.3; HL 8.9; IOD 3.1; IND 1.8; EW 2.6; FL 14.1; ED 2.7; E-N 1.9; TL/SVL 0.44; FL/SVL 0.54; HL/SVL 0.34;

In life, dorsal coloration of head, body, and limbs pale brown with pale and dark markings that include a dark brown interorbital bar with a cream border in the anterior margin, a brownish-cream V-shaped fold with a dark brown border in the posterior margin, one dark brown chevron with brownish-cream borders in the middle of the dorsum, one diagonal stripe on the flanks with a pale cream border, and two transverse bars on the hind limbs; dark brown head markings include a bold canthal and supratympanic stripes with cream borders, and a bold labial bar below the eyes with a cream border in the posterior margin (Fig. 2A); groin, anterior and posterior surface of thighs, and concealed surface of shanks red; ventral surface yellowish-brown with palms, soles and ventral surface of thighs brown (Fig. 2B); iris dark or light bronze with fine black reticulations and a faint reddish stripe across the middle.

In preservative, dorsum of head, body, limbs, and sides of head grayish-brown with the same dark brown markings and the same pale borders (now grayish-cream) such as described above; groin, anterior and posterior surface of thighs, and concealed surface of shanks grayish-brown; ventral surface on throat and chest pale tan, belly grayish-cream, with palm, soles, and limbs brown (Fig. 3).

### Variation

Sexual dimorphism is evident in respect to snout-vent length, with males smaller than females: SVL 11.3–17.4 mm in males and 19.4–27.1 mm in females (Table 1). In life, the ventral coloration of males and females can vary from brownish-cream to yellowish-cream (Figs. 4C, 4F, and 4J); only one male (CORBIDI 7381) has a grayish venter with dark brown flecks in the throat and belly (Fig. 4H). The dorsal coloration is variable: one female (CORBIDI 7380) has a dull brown dorsum without distinct markings except for a dark brown interorbital bar (Fig. 4A); one female (CORBIDI 7383) has a narrow pale middorsal stripe (Fig. 4E); one male (CORBIDI 7381) has a grayish-brown dorsum with dark brown blotches, a black labial bar below the eyes and lack canthal stripe (Fig. 4G); one male (CORBIDI 7381) has dark yellow flanks (Fig. 4L); one female (CORBIDI 7387) has an orange hue on dorsum with the markings similar to those of the holotype except for the canthal stripe (Fig. 4K); one male (CORBIDI 7386) has a distinct black canthal and supratympanic stripe (Fig. 4I).

### Distribution and natural history

**Figure 5:**
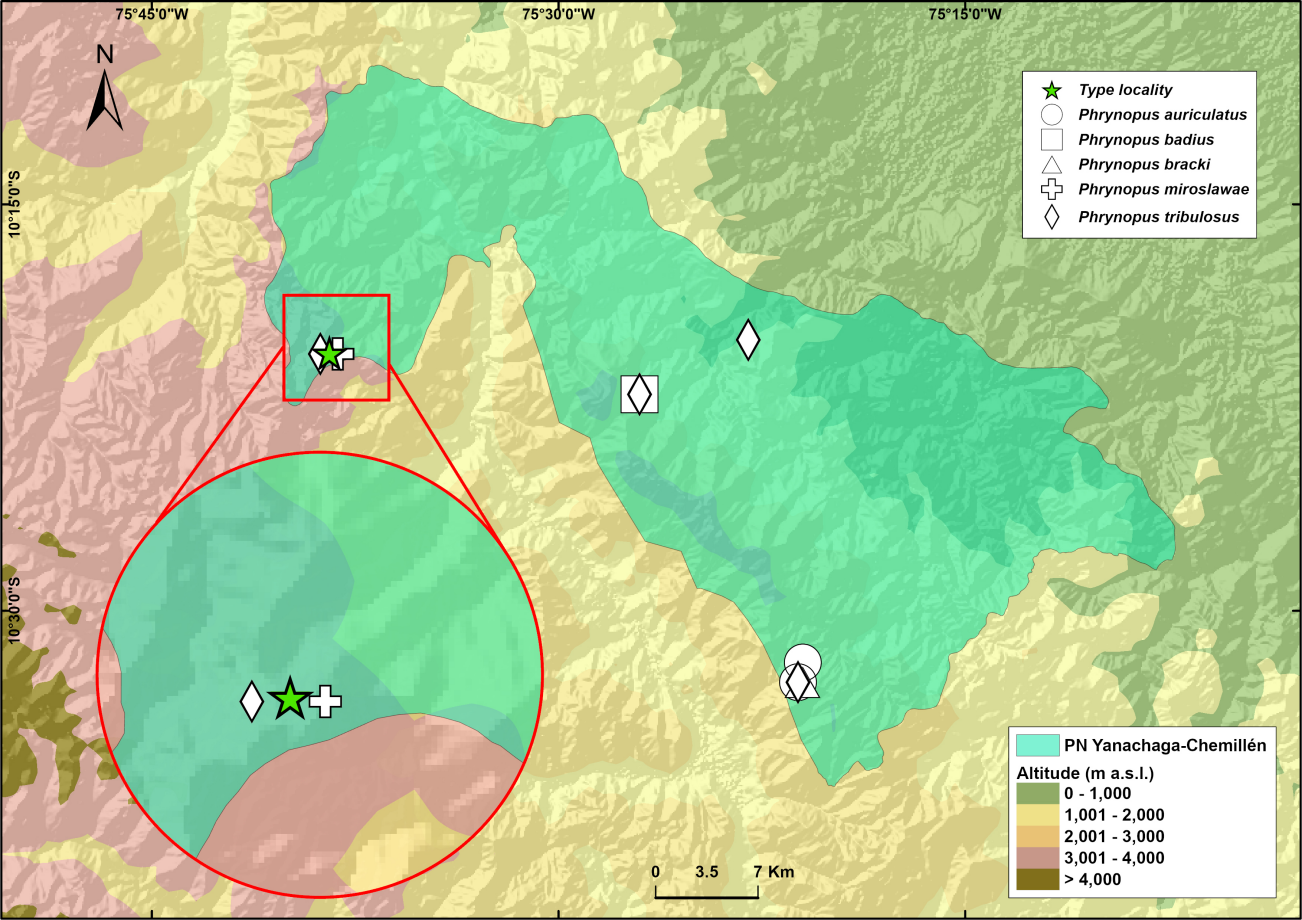
Map of the National Park Yanachaga Chemillen showing the distribution of all species of *Phrynopus* that inhabit within the park limits. The type locality of *Phrynopus manuelriosi* sp. nov. new species and *P. melanoinguinis* sp. nov. is represented by a star.

*Phrynopus manuelriosi* sp. nov. is only known from the type locality in the west margin of Río Huancabamba at an elevation of 3,280 m a.s.l., Provincia de Oxapampa, Departamento de Pasco, on the eastern slope of Cordillera Oriental in central Peru (Fig. 5). Eighteen individuals, of which ten were collected, were found in 5 hours surveying amphibians at night by four collectors. All individuals were found on the ground in the elfin forest, and perched on leaves and branches about 20–100 cm above the ground in the forest and in the riparian vegetation. The herpetological survey in Santa Bárbara occurred in the dry season and no rainfall was recorded during the four days of survey in this locality. The two new species described herein were found in sympatry in the elfin forest of Santa Bárbara. *Gastrotheca griswoldi* also occurs in the area, but above tree line in the Puna grasslands. Two other species of *Phrynopus* are known to occur in the same area of Santa Bárbara: *P. miroslawae* and *P. tribulosus*, although we did not observe them during our surveys. *Phrynopus miroslawae* is found in the elfin forest and might be syntopic with *P. manuelriosi* sp. nov., whereas *P. tribulosus* inhabits the Puna grasslands.

### Etymology

The name is a patronym for Manuel Ríos, a Peruvian forest engineer and professor at the Faculty of Forestry at Universidad Nacional Agraria La Molina (UNALM), Lima, Peru, from 1970 to 2017, who has dedicated his life to preserving the natural heritage of his country. As professor, Manuel trained hundreds of students, inspiring them to become committed advocates for resource conservation, wildlife management and the protection of natural areas. He is also a founder and life member of the Board of Directors of the Peruvian Foundation for the Conservation of Nature (Pro Naturaleza), an organization that has played a key role in the preservation and protection of the environment in Peru. Likewise, he was Director of the Conservation Data Center (CDC-UNALM) between 1983 and 1998, and his legacy is present in the creation and planning of some of the most emblematic protected areas in Peru: the Paracas National Reserve, the Titicaca National Reserve, the Lachay National Reserve, the Abiseo National Park, and the Tabaconas Namballe National Sanctuary, among many others.

**Table:** 

**Figure 6:**
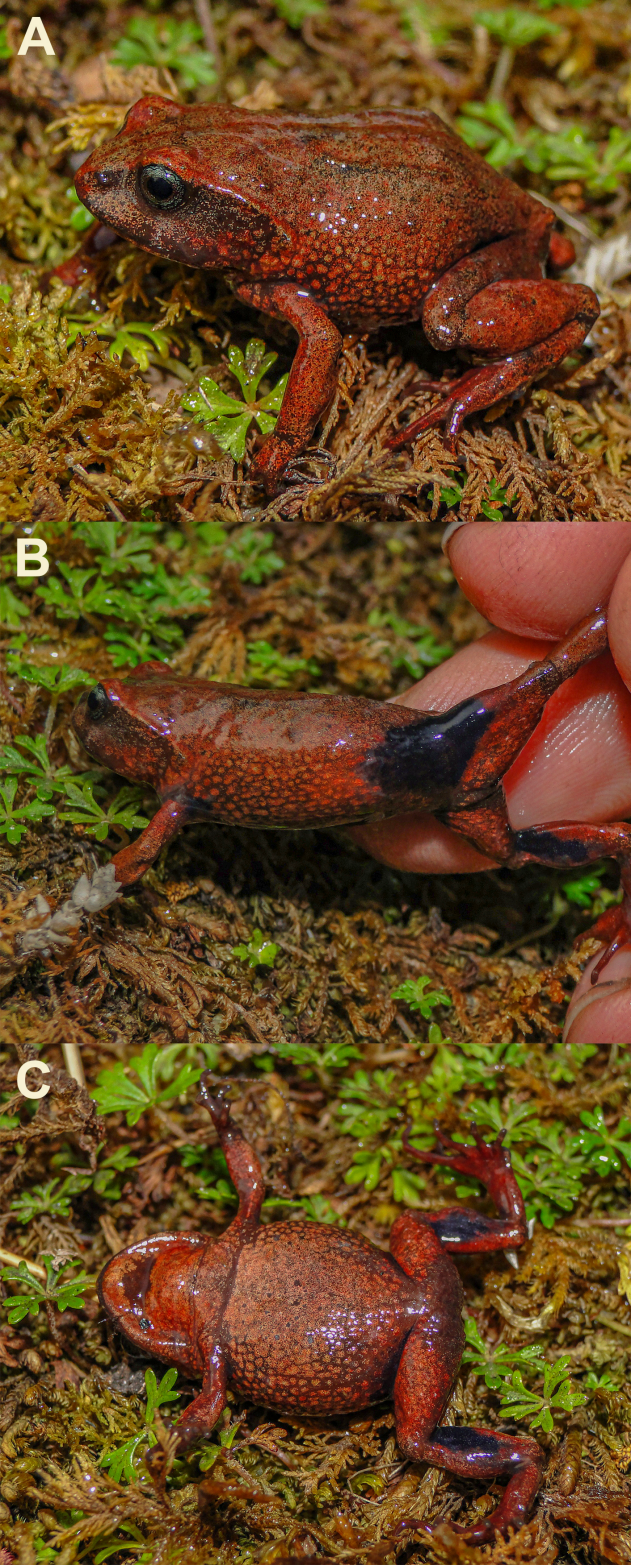
Holotype of *Phrynopus melanoinguinis.* sp. nov. (CORBIDI 7379) in life. (A) Dorsolateral, (B) lateral (showing the characteristic black coloration on the groin), and (C) ventral views (SVL 23.6 mm).

**Figure 7:**
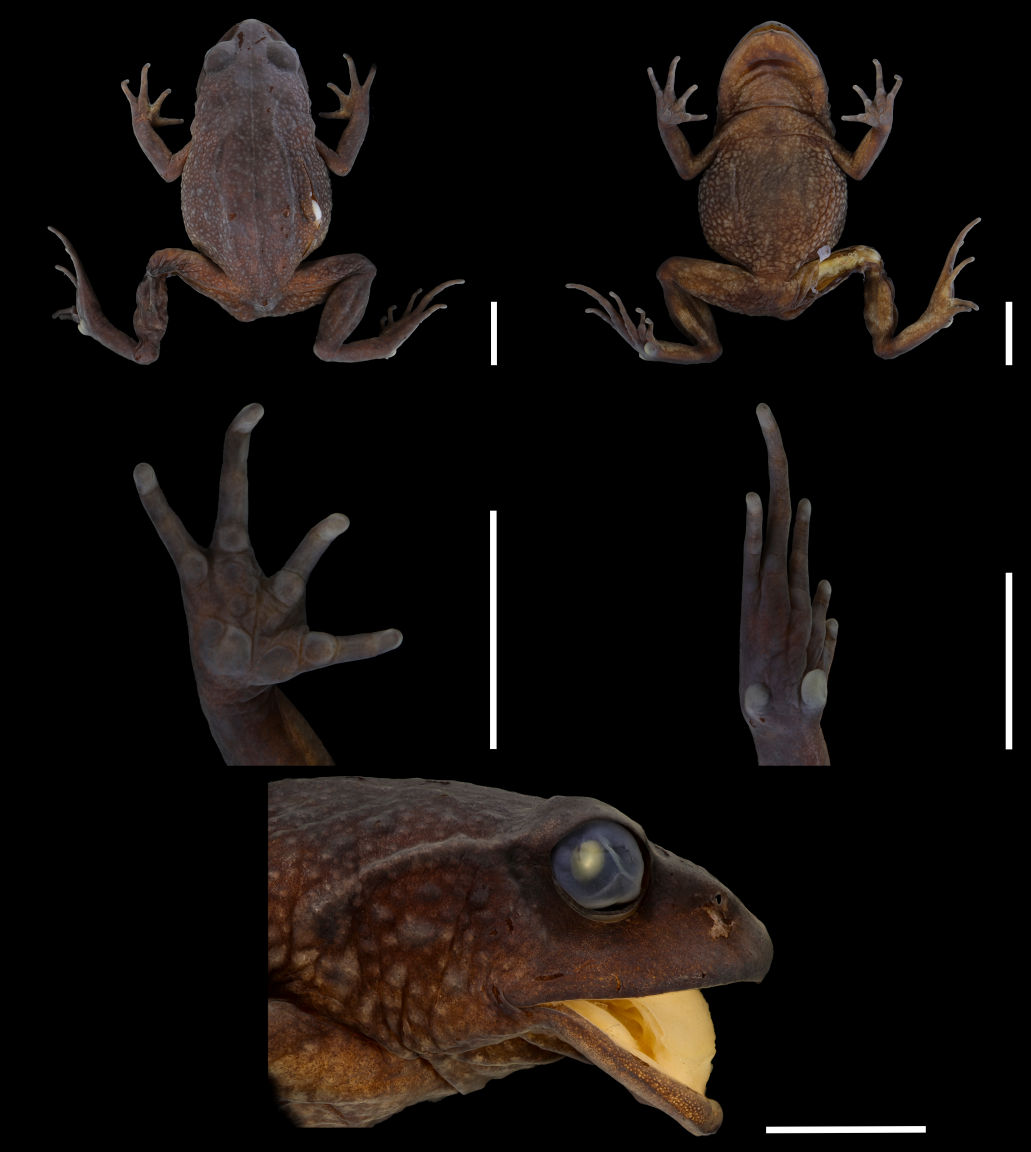
Preserved holotype of *Phrynopus melanoinguinis* sp. nov. (A) Dorsal view, (B) ventral view, (C) palm, (D) sole, and (E) head in lateral view. Scale five mm. Photographs by LAGA.

***Phrynopus melanoinguinis*** sp. nov.**** urn:lsid:zoobank.org:act:13A87303-F889-41BD-A67E-F00D4C611F17
Figs. 6–7

### Holotype

CORBIDI 7379 (Fig. 6), adult female, Santa Bárbara, Distrito de Huancabamba, Provincia de Oxapampa, Departamento de Pasco, Peru (10°20′29.1″S, 75°38′27.1″W, 3,280 m a.s.l.), collected by Pablo J. Venegas on 25 August 2010.

### Diagnosis

A species in the genus *Phrynopus* characterized by (1) skin on dorsum smooth with scattered granules; flanks and venter areolate; dorsolateral folds present, short; discoidal fold present only as thoracic fold; supratympanic fold conspicuous and long, slightly curved above the tympanic region; (2) tympanic membrane and annulus absent; (3) snout moderately short, bluntly rounded in dorsal view and in profile; (4) upper eyelids narrower than IOD, bearing low small tubercles; cranial crests absent; (5) vomerine teeth absent; (6) males unknown; (7) Finger I shorter than Finger II; tips of fingers rounded and narrow; (8) fingers lacking lateral fringes; subarticular tubercles small, rounded, weakly defined in dorsal view and flat in lateral view; supernumerary tubercles present, weakly defined; (9) ulnar tubercles absent; (10) heel and outer edge of tarsus lacking tubercles; inner tarsal fold absent; (11) inner metatarsal tubercle ovoid, prominent, about equal in size to lower, rounded, outer metatarsal tubercle; subarticular tubercles small, round, distinct only at the base of toes in dorsal view and flat on lateral view; supernumerary plantar tubercles absent; (12) toes lacking lateral fringes; webbing absent; Toe V slightly longer than Toe III; tips of toes rounded; (13) in life, dorsum dark brown without marks; groin, anterior and posterior surface of thighs, and concealed surface of shanks black; ventral surface brown with a black blotch on the throat; iris bluish-gray with fine black reticulation; (14) SVL of single female 23.6 mm.

### Comparisons

*Phrynopus melanoinguinis* sp. nov. is strikingly different from any other known species in the genus by having the groin and the hidden surfaces of the hind limbs black, and blueish-gray iris in life. *Phrynopus melanoinguinis* sp. nov. occurs in sympatry with *P. manuelriosi* sp. nov., *P. miroslawae,* and *P. tribulosus*, and it differs from those by lacking heel and tarsal tubercles, and by having dorsolateral and supratympanic folds (absent in *P. manuelriosi* sp. nov. and *P. tribulosus*). Three other species of *Phrynopus* occur within the Yanachaga-Chemillén National Park: *P. auriculatus*, *P. badius*, and *P. bracki* (Chaparro, Padial & De la Riva, 2008; Duellman & Lehr, 2009; Lehr & Oroz, 2012). *P. melanoinguinis* sp. nov. differs from those species by lacking heel and tarsal tubercles (heel tubercle present in *P. auriculatus* and *P. bracki*; tarsal tubercle present in *P. bracki*), by having eyelid tubercles (absent in *P. auriculatus*, *P. badius*, and *P. bracki*), and by having dorsolateral folds (absent in *P. bracki*) and supratympanic folds (absent in *P. badius*, and *P. bracki*).

The absence of heel and tarsal tubercles also distinguishes *P. melanoinguinis* sp. nov. from *P. daemon, P. dagmarae, P. interstinctus, P. vestigiatus, P. kotosh, P. oblivius, P. remotum*, and *P. unchog*. The combination of dorsolateral and long supratympanic folds also differentiates *P. melanoinguinis* sp. nov. from many other species in the genus: *P. barthlenae*, *P. bufoides*, *P. capitalis*, *P. chaparroi*, *P. daemon*, *P. dagmarae*, *P. heimorum*, *P. interstinctus*, *P. inti*, *P. juninensis*, *P. kauneorum*, *P. lapidoides*, *P. lechriorhyncus*, *P. montium*, *P. oblivious*, *P. peruanus*, *P. pesantesi*, *P. remotum*, *P. sancristobali*, *P. tautzorum*, *P. thompsoni*, *P. valquii*, and *P. vestigiatus*. In the case of *P. bufoides* and *P. sancristobali*, both species are also easily distinguished from *P. melanoinguinis* sp. nov. by the presence of conspicuous large round or elongate pustules on dorsum and flanks (dorsum smooth with areolate flanks in the new species). Moreover, the absence of tympanic membrane and annulus distinguishes the new species from *P. auriculatus*, *P. peruanus* and *P. mariellaleo* (tympanic membrane and annulus present).

*Phrynopus paucari* differs from *P. melanoinguinis* sp. nov. by having larger subconical tubercles forming discontinuous longitudinal ridges dorsolaterally (dorsal skin smooth) and venter greenish yellow with brown reticulation (dull brown). *Phrynopus pesantesi* differs from *P. melanoinguinis* sp. nov. by having ulnar tubercles (absent) and the venter brown with gray mottling (dull brown). *Phrynopus horstpauli* differs from *P. melanoinguinis* sp. nov. by having the skin of dorsum slightly tuberculate (smooth), Toe V much longer than Toe III (Toe V slightly longer than Toe III), and venter cream with brown blotches (dull brown). *Phrynopus barthlenae*, *P. heimorum* and *P. tautzorum* differ from *P. melanoinguinis* sp. nov. by having Toe III larger than Toe V, while Toe III is shorter than Toe V in *P. melanoinguinis* sp. nov. Furthermore, the dorsum is coarsely tuberculate in *P. barthlenae* and *P. apumantarum*, and smooth in *P. melanoinguinis* sp. nov. In *P. apumantarum*, the skin on the entire venter is coarsely areolate, while in *P. melanoinguinis* sp. nov. is areolate peripherally and smooth in the center. *Phrynopus kauneorum* and *P. lechriorhynchus* differ from *P. melanoinguinis* sp. nov. by the presence of dentigerous processes of vomers, whereas these are absent in *P. melanoinguinis* sp. nov.; in addition, *P. kauneorum* lacks dorsolateral fold (dorsolateral fold present in the new species), while *P. lechriorhynchus* has the snout spatulate, long and depressed, broadly rounded in dorsal view and sloping anteroventrally in profile (snout short and bluntly rounded in dorsal view and in profile in *P. melanoinguinis* sp. nov.).

In addition, *P. personatus* from the Río Abiseo National Park (Departamento San Martín) in northern Peru is similar to *P. melanoinguinis* sp. nov. in that both species have the groins and hidden surfaces of hind limbs black ( Rodríguez & Catenazzi, 2017). However, *P. personatus* has the black surfaces of groins and hind limbs adorned by conspicuous white blotches and the skin of dorsum shagreen with scattered tubercles (dorsum smooth in *P. melanoinguinis* sp. nov.).

### Description of the holotype

Adult female (Figs. 6 and 7); body moderately robust; head narrower than body, nearly as long as wide; snout bluntly rounded in dorsal view and in profile; canthus rostralis slightly curved in dorsal view, rounded in profile; loreal region nearly flat; lips rounded; nostrils barely protuberant, directed laterally; internarial region flat; top of head flat; width of upper eyelid narrower than IOD (EW/IOD 0.74); eye large, its diameter greater than its distance from nostril (E-N/ED 0.63); tympanic membrane and annulus, absent; supratympanic fold conspicuous and long, slightly curved above the tympanic region; ovoid postrictal tubercles present, minute. Tongue slightly longer than broad, not notched posteriorly, posterior half free; choanae small, rounded, not concealed by palatal shelf of maxillary; dentigerous processes of vomers absent.

Forelimb slender; ulnar tubercles absent; palmar tubercle low, round, slightly longer than thenar tubercle; subarticular tubercles small and rounded in dorsal view, and flat on lateral view; two supernumerary tubercles present, weakly defined; fingers short and slender, lacking lateral fringes; relative lengths of fingers I < II < IV < III; tips of fingers narrow, rounded, lacking circumferential grooves. Hind limb slender; heel and tarsus lacking tubercles; inner tarsal fold absent; inner metatarsal tubercle prominent, round, about twice as much of round outer metatarsal tubercle; subarticular tubercles small, rounded, weakly defined in dorsal view and flat in lateral view; supernumerary tubercles absent; toes slender, lacking lateral fringes; relative lengths of toes I < II < III < V < IV; tips of toes narrow, rounded, lacking circumferential grooves. Skin on dorsum smooth with scattered low and round tubercles, flanks areolate, and hind limbs tuberculate; dorsolateral fold present and short; upper eyelids bearing low small tubercles; skin of throat and chest areolate, on belly weakly areolate in the center and coarsely areolate peripherally; discoidal fold absent, thoracic fold present; skin ventral and ventrolateral to cloaca granular.

Measurements (in mm) and proportions of holotype: SVL 23.6; TL 8.9; HW 8.7; HL 9.1; IOD 2.7; IND 1.8; EW 2; FL 9.3; ED 2.7; E-N 1.7; TL/SVL 0.38; FL/SVL 0.39; HL/SVL 0.39; HW/SVL 0.37; HW/HL 0.96; E-N/ED 0.63; EW/IOD 0.75.

In life, dorsal surface of head, body and limbs, and flanks reddish-brown (Fig. 6A); anterior and posterior surface of thighs, groin, and concealed surface of tibia black (Fig. 6B); ventral surface pale reddish-brown, with a dark brown blotch on the throat (Fig. 6C); iris bluish-gray with fine black reticulation.

In preservative, dorsum dark brown without marks; groin, anterior and posterior surface of thighs and concealed surface of shanks black; ventral surface brown with a black blotch on the throat (Fig. 7).

### Distribution and natural history

*Phrynopus melanoinguinis* sp. nov. is known only from the type locality and is syntopic with *P. manuelriosi* sp. nov. The type locality for both new species described here is within the Peruvian Yanachaga-Chemillén National Park (Fig. 5). The single specimen collected was found on a mossy ground at night in the elfin forest habitat. *Phrynopus melanoinguinis* sp. nov. might be syntopic with *P. miroslawae* and sympatric with *P. tribulosus*.

### Etymology

The specific name is an adjective derived from the Greek *melano* (meaning black) and the Latin *inguinis* (meaning groin) and is used as a noun in apposition. The name refers to the species’ distinctive black groin.

## Discussion

Most species of *Phrynopus* have a cryptic mode of life restricted to leaf litter and moss layers, making it difficult to find and observe individuals in the field (Lehr & Oroz, 2012). Consequently, many species are known from a limited number of specimens, reflecting the rarity of some species in this genus (Lehr & Oroz, 2012; Rodríguez & Catenazzi, 2017), which leads to descriptions based on a few or even a single specimen, including our description of *P. melanoinguinis* sp. nov. The challenge of describing a species based on few specimens is related to the lack of understanding of the intraspecific variation in diagnostic characters (Köhler & Padial, 2016). However, that can be mitigated by using multiple lines of evidence (*e.g.*, morphological and molecular in the case of *P. melanoinguinis* sp. nov.) to name well-supported singleton species.

While *Phrynopus melanoinguinis* sp. nov. is known only from the female holotype, the type series of *P. manuelriosi* sp. nov. is comparatively large (five females and five males). Limited number of male specimens seems to be recurrent in *Phrynopus*, as for three other species only females are known (*P. miroslawae*, *P. thompsoni* and *P. vestigiatus*), while in eight species only one male has been collected (*P. lapidoides*, *P. unchog*, *P. anancites*, *P. capitalis*, *P. personatus*, *P. daemon* and *P. chaparroi*). This broader pattern across the genus could represent actual female-to-male ratios in populations, but could also be related to natural history aspects of the species. Even though little is known about the reproductive behavior of most species of *Phrynopus*, the “secretiveness of males” can explain why males are harder to find (Lehr, 2001). For example, males of *P. bracki* have been reported to call from hidden places in leaf litter and moss vegetation (Hedges, 1990) and males of *P. peruanus* were found calling from inside grass tussocks (Chaparro et al., 2007). Moreover, males of *P. badius* and *P. tribulosus* were heard calling, but could not be located in the dense vegetation, despite the collectors’ efforts (Lehr, Moravec & Cusi, 2012).

The occurrence of two or more species of *Phrynopus* in the same locality was considered rare, but recent surveys have reported many cases where species of *Phrynopus* co-occur in the same region (see Chaparro, Padial & De la Riva, 2008; Chávez et al., 2015; Duellman & Hedges, 2008; Lehr, 2001; Lehr & Aguilar, 2002; Lehr, Aguilar & Köhler, 2002; Lehr, Fritzsch & Müller, 2005; Lehr, Moravec & Cusi, 2012; Lehr & Oroz, 2012; Lehr & Rodríguez, 2017; Rodríguez & Catenazzi, 2017; Von May, 2017). In many cases, species segregate by elevation or by microhabitat type (Rodríguez & Catenazzi, 2017). *Phrynopus manuelriosi* sp. nov. and *P. melanoinguinis* sp. nov. were found in sympatry in the elfin forest at 3,280 m elevation, but apparently show some microhabitat segregation, since only *P. manuelriosi* sp. nov. is known to perch on leaves and branches about 20–100 cm above the ground in the forest and in the riparian vegetation.

**Figure 8:**
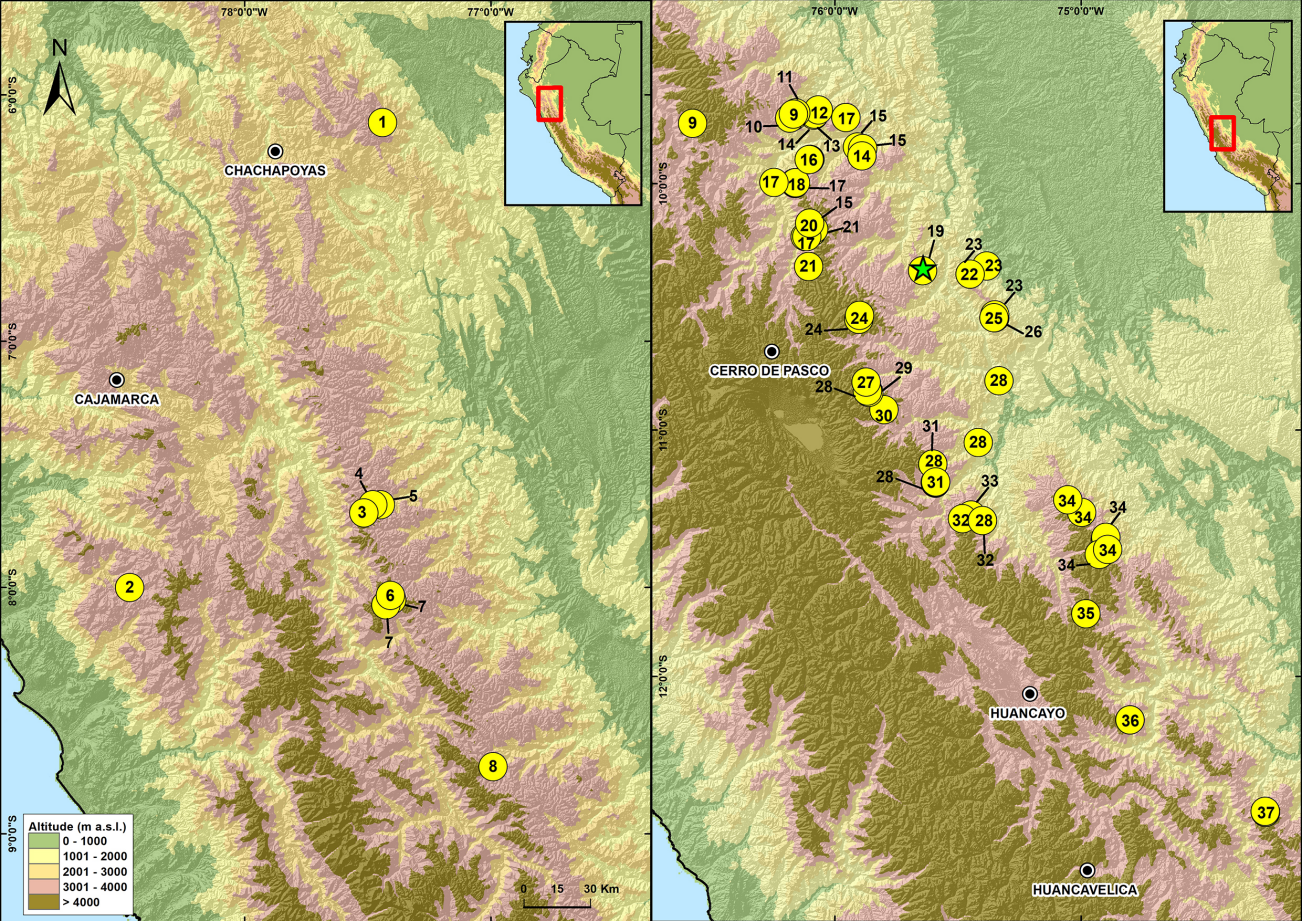
Map of Peru showing the distribution of species in the genus *Phrynopus*. 1. *P. mariellaleo*; 2. *P. thompsoni*; 3.*P. capitalis*; 4.*P. dumicola*; 5.*P. personatus*; 6. *P. anancites*; 7. *P. valquii*; 8. *P. remotum*; 9.*P. daemon*; 10. *P. lapidoides*; 11. *P. unchog*; 12. *P. vestigiatus*; 13. *P. lechriorhynchus*; 14. *P. kauneorum*; 15. *P. dagmarae*; 16. *P. interstinctus*; 17. *P. horstpauli*; 18. *P. heimorum*; 19.*P. miroslawae*; 20. *P. tautzorum*; 21. *P. barthlenae*; 22. *P. badius*; 23. *P. tribulosus*; 24. *P. pesantesi*; 25.*P. auriculatus*; 26. *P. bracki* ; 27.*P. paucari*; 28. * P. juninensis*; 29. *P. bufoides*; 30. *P. kotosh*; 31. *P. montium*; 32. *P. peruanus* ; 33. *P. oblivius*; 34. *P. inti*; 35*.P. chaparroi*. 36. *P. apumantarum*. 37. *P. sancristobali*. The green star represents the type locality of *P. manuelriosi* sp. nov. and *P. melanoinguinis* sp. nov.

These findings support previous hypotheses about high levels of microendemism and beta-diversity in Andean amphibians (Fig. 8), and the region likely has many species still to be discovered (Rodríguez & Catenazzi, 2017). The addition of the two new species described herein also increases the number of species of *Phrynopus* known from Cordillera Yanachaga to seven (*Phrynopus auriculatus*, *P. badius, P. bracki, P. manuelriosi* sp. nov., *P. melanoinguinis* sp. nov.*, P. miroslawae,* and *P. tribulosus*). This region shows the highest regional species diversity of *Phrynopus*, along with Cordillera de Carpish (*P. daemon*, *P. dagmarae*, *P. interstinctus*, *P. kauneorum*, *P. lapidoides*, *P. unchog*, and *P. vestigiatus*), and followed by Río Abiseo National Park (*P. anancites*, *P.capitalis*, *P. dumicola*, *P. personatus*, *P. valquii*). Moreover, Santa Bárbara is the only case of a type locality shared by three species of *Phrynopus* (*P. manuelriosi* sp. nov., *P. melanoinguinis* sp. nov., and *P. miroslawae*). The sympatric *P. auriculatus* and *P. bracki* occur 38 km airline SE of Santa Bárbara, 5.5 km E Oxapampa 2,600 m, on mountains at the opposite side of the Oxapampa valley (Chaparro, Padial & De la Riva, 2008), while *P. badius* is closer at the eastern margin of Río Huancabamba (20 km airline SE of Santa Bárbara). Santa Bárbara is located in the northwestern extreme of the Peruvian Yanachaga-Chemillén National Park (see Fig. 5) and, like in other regions throughout the Peruvian Andes, montane habitats are continuously destructed due to the increase of agricultural land and cattle ranching (Dillon et al., 1995; Venegas, 2007; Weigend, Rodríguez & Arana, 2005), which constitutes a serious threat to the species occurring therein. During our survey of amphibians and reptiles for four days in Santa Bárbara, we recorded several forest fires in the buffer zone of Yanachaga-Chemillén National Park, and we also found remains of forest fire in the grasslands and at the tree line within the national park limits (an area deemed banned for resource use). Although both species are present within the park, evidence of habitat destruction in the area raises concerns about whether these species are truly protected. Many Peruvian species of high Andean strabomantid frogs in the genera *Bryophryne, Qosqophryne, Psychrophrynella, Phrynopus*, and some *Pristimantis* have highly restricted ranges; hence, it is unlikely to find the new species of *Phrynopus* described herein in other parts of the national park or its surroundings. Although we agree that it is important to keep records of species that are included in threatened categories lists (*e.g.*, International Union for Conservation of Nature (IUCN) Red List, Peruvian government threatened species list) or in natural protected areas (Aguilar et al., 2010; Von May et al. 2008), we believe that the fact that these species are legally protected will not grant their survival, especially if there are no means to secure the park boundaries from intrusions. This needs to be considered when classifying these frogs in threat categories or evaluations for becoming a priority for protection in governmental conservation plans.

## Conclusions

We describe two new species of the Andean genus *Phrynopus*, *P. manuelriosi* sp. nov. and *P. melanoinguinis* sp. nov., based on robust morphological and molecular evidence. The new species occur sympatrically in the Andean elfin forest at an elevation of 3,280 m, in the Yanachaga-Chemillen National Park in Departamento de Pasco, Peru. Two other species of the genus, *P. miroslawae* and *P. tribulosus,* are also known to occur in the same locality. *Phrynopus melanoinguinis*, *P. miroslawae* and *P. tribulosus* are terrestrial, as is typical for most members of the genus, whereas *P. manuelriosi* possesses arboreal habitus. Despite their discovery within the boundaries of a national park, the long-term survival of these new species is not guaranteed if the borders of the protected area are not well protected. Fires observed both within the park and in its buffer zone underscore the urgent need for effective protection measures, particularly given the restricted distribution ranges characteristic of *Phrynopus* species. This highlights the vulnerability of these frogs and the critical importance of conserving their fragile habitats.

## Supplemental Information

10.7717/peerj.20250/supp-1 Supplemental Information 1 Primers used to amplify mitochondrial and nuclear genes fragments

10.7717/peerj.20250/supp-2 Supplemental Information 2 Specimens examined in this study

10.7717/peerj.20250/supp-3 Supplemental Information 3 GenBank accession numbers of species sampled in this study

10.7717/peerj.20250/supp-4 Supplemental Information 4 Sequences used in the phylogenetic analysis and submitted to GenBank

10.7717/peerj.20250/supp-5 Supplemental Information 5 Partitions

10.7717/peerj.20250/supp-6 Supplemental Information 6 Alignment

10.7717/peerj.20250/supp-7 Supplemental Information 7 Script

## Additional Information and Declarations

### Field Study Permissions

The following information was supplied relating to field study approvals (i.e., approving body and any reference numbers): We obtained our research permit through the Dirección General Forestal y de Fauna Silvestre, Ministerio de Agricultura y Riego, Peru, which issued the Contrato de Acceso Marco a Recursos Genéticos. Approval number: 359-2013-MINAGRI-DGFFS-DGEFFS.

### DNA Deposition

The following information was supplied regarding the deposition of DNA sequences: The sequences are available at GenBank: PV034806, PV036306, PV230486, PV230487, PV034827, PV034805, PV036305, PV230484, PV230485, PV034826, PV036304, PV230482, PV230483, PV034825, Phrynopus sp. ma AM-2025, Phrynopus sp. me AM-2025.

### Data Availability

The following information was supplied regarding data availability: The alignments, script, and output files for the phylogenetic analysis, including partitions and trees are available in the Supplemental Files.

### New Species Registration

The following information was supplied regarding the registration of a newly described species: Publication LSID: urn:lsid:zoobank.org:pub:513EDEF4-3F07-4097-8902-8DFED468E4C3, *Phrynopus manuelriosi* LSID: urn:lsid:zoobank.org:act:86453AE1-9A6A-4ABD-9681-CF9E372B877D *Phrynopus melanoinguinis* LSID: urn:lsid:zoobank.org:act:13A87303-F889-41BD-A67E-F00D4C611F17.
