# Supplementary material for: Two new sympatric species of Phrynopus (Anura: Strabomantidae) from the Elfin Forests of Cordillera de Yanachaga in central Peru
Source: PeerJ. 2025 Oct 30;13:e20250. doi: 10.7717/peerj.20250 (PMC12579853; doi:10.7717/peerj.20250)
Supplement: Supplemental Information 1 [file peerj-13-20250-s001.docx]

**Table S1**. Primers used to amplify mitochondrial and nuclear genes fragments.

| **Gene fragment** | **Primer name** | **Direction** | **Primer sequence (5' to 3')** | **Reference** |
| --- | --- | --- | --- | --- |
| 12S-tRNAval-16S | MVZ59 | Forward | ATAGCACGTAAAAYGCTDAGATG | Graybeal, 1997 |
|  | tRNA-VAl | Reverse | GGTGTAAGCGARAGGCTTTKGTTAAG | Goebel et al., 1999 |
|  | 12S-L13 | Forward | TTAGAAGAGGCAAGTCGTAACATGGTA | Feller and Hedges, 1998 |
|  | 16S-H10 | Reverse | TGCTTACGCTACCTTTGCACGGT | Hedges, 1994 |
|  | 16Sa-L | Forward | CGCCTGTTTATCAAAAACAT | Palumbi et al., 2002 |
|  | 16Sb-H | Reverse | CCCGTCTGAACTCAGATCACGT | Palumbi et al., 2002 |
| COI | AnF1 (T3) | Forward | ACHAAYCAYAAAGAYATYGG | Lyra et al., 2017 |
|  | AnR1 (T7) | Reverse | CCRAARAATCARAADARRTGTTG | Lyra et al., 2017 |
| RAG-1 | R1-GFF | Forward | GAGAAGTCTACAAAAAVGGCAAAG | Faivovich et al., 2005 |
|  | R1-GFR | Reverse | GAAGCGCCTGAACAGTTTATTAC | Faivovich et al., 2005 |
| TYR | Tyr1C | Forward | GGCAGAGGAWCRTGCCAAGATGT | Bossuyt and Milinkovitch, 2000 |
|  | Tyr1G | Reverse | TGCTGGCRTCTCTCCARTCCCA | Bossuyt and Milinkovitch, 2000 |

**References for this section:**

**Bossuyt, F., and M. C. Milinkovitch.** 2000. Convergent adaptive radiations in Madagascan and Asian ranid frogs reveal covariation between larval and adult traits. Proceedings of the National Academy of Sciences USA 97:6585–6590.

**Faivovich, J., C. F. B. Haddad, P. C. A. Garcia, D. R. Frost, J. A. Campbell, and W. C. Wheeler.** 2005. Systematic review of the frog family Hylidae, with special reference to Hylinae: phylogenetic analysis and taxonomic revision. Bulletin of the American Museum of Natural History 294:1–240.

**Feller, A. E., S. B. and Hedges.** 1998. Molecular evidence for the early history of living amphibians. Molecular Phylogenetics and Evolution 9:509–516.

**Graybeal, A.** 1997. Phylogenetic relationships of bufonid frogs and tests of alternate macroevolutionary hypothesis characterizing their radiation. ‎ Zoological Journal of the Linnean Society 119:297–338.

**Goebel, A. M., J. M. Donnelly, and M. E. Atz.** 1999. PCR primers and amplification methods for 12S ribosomal DNA, the control region, cytochrome oxidase I, and cytochrome b in bufonids and other frogs, and an overview of PCR primers which have amplified DNA in amphibians successfully. Molecular Phylogenetics and Evolution 11:163–199.

**Lyra, M. L., C. F. B. Haddad, A. M. L. de Azeredo-Espin.** 2017. Meeting the challenge of DNA barcoding Neotropical amphibians: polymerase chain reaction optimization and new COI primers. Molecular Ecology Resources 17:966–980.

**Palumbi, S. R., A. Martins, S. Romano, W. O. Mc-Millan, L. Stice, and G. Grabawski.** 2002. The Simple fool’s guide to PCR, version 2.0. Department of Zoology and Kewalo Marine Laboratory, University of Hawaii, Honolulu. Privately published, compiled by S. Palumbi.
