# Supplementary material for: Two new sympatric species of Phrynopus (Anura: Strabomantidae) from the Elfin Forests of Cordillera de Yanachaga in central Peru
Source: PeerJ. 2025 Oct 30;13:e20250. doi: 10.7717/peerj.20250 (PMC12579853; doi:10.7717/peerj.20250)
Supplement: Supplemental Information 2 [file peerj-13-20250-s002.docx]

**Appendix 1. Specimens examined in this study.**

*Noblella duellmani*: MUSM 19856; *Phrynopus auriculatus*: CORBIDI 10304, KU 291633, 291634; *Phrynopus badius*: MUSM 31099; *Phrynopus barthlenae*: MUSM 20606, 20603, 20603, 20604, 20605, 20608, 20609, CORBIDI 10302–03; *Phrynopus bracki*: CORBIDI 10302–03; *Phrynopus bufoides*: MUSM 18074 (20358), 31246; *Phrynopus capitalis*: AMNH 134158; *Phrynopus chaparroi*: MHNCP 10983, 10982, 10984, 10985, 10981; *Phrynopus curator*: MUSM 31106; *Phrynopus daemon*: CORBIDI 15364; *Phrynopus dagmarae*: MUSM 20451, 20449, 20580, 20456, 20579, 20453, 20448, 20456, KU 196592, CORBIDI 14552–59; *Phrynopus dumicola*: AMNH 134149, 134152; *Phrynopus heimorum*: MUSM 20441, 20442, 20443, 20444, 20446; *Phrynopus horstpauli*: MUSM 20424, 20437, 20435, 20434, 20426, 20436, 20435, 20430, 20439, KU 291400, 311453, CORBIDI 16662–3; *Phrynopus interstinctus*: MUSM 29543, 29544, 29545; *Phrynopus juninensis*: MCZ 22851, 24361; *Phrynopus kauneorum*: MUSM 20459, 20451, KU 311451; *Phrynopus lapidoides*: MUSM 32750; *Phrynopus mariellaleo*: CORBIDI 11668, 11669, 11657, 11692, 11707, 11796, 11799; *Phrynopus miroslawae*: MHNCP 6469; *Phrynopus montium*: MCZ 22858, 22859; *Phrynopus oblivius*: MUSM 19979, 19980, 19981; *Phrynopus paucari*: MUSM 20657; *Phrynopus personatus*: AMNH 134153; *Phrynopus peruanus*: MUSM 19977, 19978, MCZ 24318, 24311, 24319; *Phrynopus pesantesi*: MUSM 19857, 19860; *Phrynopus tautzorum*: MUSM 20613; *Phrynopus thompsoni*: KU 288712; *Phrynopus tribulosus*: KU 291630; *Phrynopus tribulosus*: MHNCP 6441, CORBIDI 10299–301; *Phrynopus unchog*: MUSM 32749, 32748; *Phrynopus valquii*: CORBIDI 14005, 14007, 13988, 13995, 13998-14001, 14003-04, KU 220918; *Phrynopus vestigiatus*: MUSM 29542, CORBIDI 14598–14602, 14617–18, 14958, 16090.
