# Supplementary material for: Two new sympatric species of Phrynopus (Anura: Strabomantidae) from the Elfin Forests of Cordillera de Yanachaga in central Peru
Source: PeerJ. 2025 Oct 30;13:e20250. doi: 10.7717/peerj.20250 (PMC12579853; doi:10.7717/peerj.20250)
Supplement: Supplemental Information 3 [file peerj-13-20250-s003.docx]

**Appendix 2:** GenBank accession numbers of species sampled in this study.

[Note to editor and reviewers: GenBank accession number for newly produced sequences will be added after acceptance of manuscript.]

| Species | 12S | 16S | RAG | TYR | COI |
| --- | --- | --- | --- | --- | --- |
| *Haddadus binotatus* | EF493361 | EF493361 | EF493397 | JX267685 | KU494450 |
| *Lynchius flavomaculatus* | EU186667 | EU186667 | EU186745 | EU186766 | - |
| *Lynchius megacephalus* | - | MK423938 | - | - | - |
| *Lynchius nebulanastes* | EU186704 | EU186704 | - | - | - |
| *Lynchius oblitus* | KX470777 | KX470784 | - | KX470800 | - |
| *Lynchius parkeri* | EU186705 | EU186705 | MK423933 | - | - |
| *Lynchius simmonsi* | JF809940 | JF810004 | JF809915 | JF809894 | - |
| *Lynchius tabaconas* | KX470773 | KX470780 | - | KX470796 | - |
| *Lynchius waynehollomonae* | - | MZ015002 | - | - | - |
| *Oreobates amarakaeri* | JF809934 | JF809996 | JF809913 | JF809891 | - |
| *Oreobates antrum* | MH025427 | MH025451 | MH025436 | MH025445 | - |
| *Oreobates ayacucho* | JF809933 | JF809970 | JF809912 | JF809890 | - |
| *Oreobates barituensis* | JF809935 | JF809999 | JF809914 | JF809892 | - |
| *Oreobates berdemenos* | - | KJ125507 | - | - | KJ125511 |
| *Oreobates chiquitanus* | JF809923 | EU192296 | JF809902 | JF809882 | - |
| *Oreobates choristolemma* | JF809921 | FJ539067 | JF809900 | JF809881 | - |
| *Oreobates discoidalis* | FJ539073 | FJ539068 | JF809904 | JF809884 | EU368883 |
| *Oreobates gemcare* | JF809930 | JF809960 | JF809909 | - | - |
| *Oreobates granulosus* | JF809929 | EU368897 | - | JF809887 | - |
| *Oreobates heterodactylus* | - | KJ125510 | - | - | - |
| *Oreobates ibischi* | FJ438817 | FJ438806 | - | - | - |
| *Oreobates lehri* | JF809927 | JF809957 | JF809906 | - | - |
| *Oreobates lundbergi* | - | JF809959 | - | - | - |
| *Oreobates machiguenga* | JF809932 | JF809969 | JF809911 | JF809889 | - |
| *Oreobates madidi* | FJ539075 | FJ539070 | JF809901 | - | EU368887 |
| *Oreobates pereger* | JF809926 | JF809955 | JF809905 | JF809885 | - |
| *Oreobates quixensis* | EF493828 | EF493662 | - | - | KU494611 |
| *Oreobates remotus* | MH025425 | JN688273 | MH025434 | MH025443 | JN688276 |
| *Oreobates sanctaecrucis* | JF809924 | JF809951 | JF809903 | JF809883 | - |
| *Oreobates sanderi* | - | EU368904 | - | - | EU368891 |
| *Oreobates saxatilis* | EU186726 | EU186708 | EU186742 | EU186763 | - |
| *Oreobates yanucu* | - | KY111322 | - | - | - |
| *Phrynopus auriculatus* | EF493708 | EF493708 | - | - | - |
| *Phrynopus auriculatus* | MF186290 | MF186348 | - | MF186582 | MF186466 |
| *Phrynopus badius* | MG896594 | MG896571 | MG896618 | - | MG896611 |
| *Phrynopus badius* | MG896595 | MG896572 | MG896619 | - | MG896612 |
| *Phrynopus barthlenae* | AM039721 | AM039653 | - | - | - |
| *Phrynopus barthlenae* | MF186292 | MF186350 | - | - | MF186464 |
| *Phrynopus bracki* | EF493709 | EF493709 | EF493421 | EF493507 | GQ345202 |
| *Phrynopus bufoides* | AM039713 | AM039645 | - | - | - |
| *Phrynopus daemon* | MG896597 | MG896574 | - | - | - |
| *Phrynopus heimorum* | AM039704 | AM039636 | - | - | - |
| *Phrynopus heimorum* | MF186302 | MF186363 | - | MF186580 | - |
| *Phrynopus horstpauli* | AM039715 | AM039647 | - | - | - |
| *Phrynopus horstpauli* | MF186303 | MF186364 | - | MF186584 | MF186465 |
| *Phrynopus interstinctus* | MG896598 | MG896575 | MG896621 | - | MG896614 |
| *Phrynopus inti* | - | MF651901 | MF651916 | - | - |
| *Phrynopus inti* | MF651909 | MF651902 | MF651917 | - | - |
| *Phrynopus inti* | MF651910 | MF651903 | - | - | - |
| *Phrynopus inti* | MF651911 | MF651904 | - | - | - |
| *Phrynopus inti* | MF651913 | MF651906 | MF651918 | MF651921 | - |
| *Phrynopus inti* | MF651914 | MF651907 | MF651919 | - | - |
| *Phrynopus inti* | MF651912 | MF651905 | - | - | - |
| *Phrynopus juninensis* | MF651915 | MF651908 | MF651920 | - | - |
| *Phrynopus juninensis* | MG896599 | MG896576 | MG896622 | - | - |
| *Phrynopus juninensis* | MG896600 | MG896577 | MG896623 | - | - |
| *Phrynopus kauneorum* | AM039718 | AM039650 | - | - | - |
| *Phrynopus kauneorum* | AM039723 | AM039655 |  |  |  |
| *Phrynopus manuelriosi* new species | PV034806 | PV036306 | PV230486 | PV230487 | PV034827 |
| *Phrynopus manuelriosi* new species | PV034805 | PV036305 | PV230484 | PV230485 | PV034826 |
| *Phrynopus mariellaleo* | - | MH538298 | MH538302 | MH538305 | - |
| *Phrynopus mariellaleo* | - | MH538299 | MH538303 | MH538306 | - |
| *Phrynopus mariellaleo* | - | MH538297 | MH538301 | MH538304 | - |
| *Phrynopus melanoinguinis* new species | - | PV036304 | PV230482 | PV230483 | PV034825 |
| *Phrynopus miroslawae* | MF186312 | MF186393 | MF186542 | MF186585 | MF186463 |
| *Phrynopus montium* | MG896601 | MG896578 | MG896624 | - | - |
| *Phrynopus montium* | MG896602 | MG896579 | MG896625 | - | - |
| *Phrynopus peruanus* | MG896603 | MG896580 | - | - | MG896615 |
| *Phrynopus peruanus* | MG896604 | MG896581 | - | - | MG896616 |
| *Phrynopus peruanus* | - | MG896582 | MG896626 | MG896631 | - |
| *Phrynopus peruanus* | - | MG896583 | MG896627 | MG896632 | - |
| *Phrynopus peruanus* | - | MG896584 | MG896628 | - | - |
| *Phrynopus peruanus* | - | MG896585 | - | - | - |
| *Phrynopus peruanus* | - | MG896586 | - | - | - |
| *Phrynopus peruanus* | - | MG896587 | - | - | - |
| *Phrynopus peruanus* | - | MG896588 | - | - | - |
| *Phrynopus pesantesi* | AM039724 | AM039656 | - | - | - |
| *Phrynopus montium* | - | MT261899 | MT431670 | MT431667 | MT263073 |
| *Phrynopus montium* | - | MT261774 | - | MT431668 | MT434010 |
| *Phrynopus montium* | MT272829 | MT261773 | MT431671 | MT431669 | MT434009 |
| *Phrynopus* sp | AM039725 | AM039657 | - | - | - |
| *Phrynopus* sp | AM039728 | AM039660 | - | - | - |
| *Phrynopus* spI | MG896606 | MG896589 | MG896629 | - | - |
| *Phrynopus* spI | MG896607 | MG896590 | MG896630 | - | - |
| *Phrynopus tautzorum* | AM039720 | AM039652 | - | - | - |
| *Phrynopus tribulosus* | MG896596 | MG896573 | MG896620 | - | - |
| *Phrynopus tribulosus* | MF186313 | MF186394 | MF186546 | MF186577 | MF186467 |
| *Phrynopus tribulosus* | EU186725 | EU186707 | - | - | - |
| *Phrynopus tribulosus* | MF186329 | MF186423 | - | MF186578 | - |
| *Phrynopus tribulosus* | MF186330 | MF186424 | MF186547 | MF186579 | MF186469 |
| *Phrynopus unchog* | MG896608 | MG896591 | - | - | - |
| *Phrynopus unchog* | MG896609 | MG896592 | - | - | - |
| *Phrynopus vestigiatus* | MG896610 | MG896593 | - | - | MG896617 |
